# Periodontal status among adolescents in Georgia. A pathfinder study

**DOI:** 10.7717/peerj.137

**Published:** 2013-09-17

**Authors:** Liran Levin, Vladimer Margvelashvili, Leon Bilder, Manana Kalandadze, Nino Tsintsadze, Eli E. Machtei

**Affiliations:** 1 Department of Periodontology, School of Graduate Dentistry, Rambam Health Care Campus, Haifa, Israel; 2 Faculty of Medicine, Technion, Israel Institute of Technology, Haifa, Israel; 3 Department of Stomatology and Maxillofacial Surgery, Faculty of Medicine, Tbilisi State University, Tbilisi, Georgia

**Keywords:** Periodontal disease, Epidemiology, Periodontitis

## Abstract

**Objectives.** The aim of the present pathfinder study was to screen and map the periodontal status of Georgian population in accordance with the guidelines of the World Health Organization for population based surveys.

**Methods.** During 2012, a pathfinder study was conducted to collect this data. For the periodontal portion of the study, 15-year-old school children were examined in the capital city of Tbilisi as well as in two other large cities and 4 smaller villages. All participants were examined by a trained dental team in a classroom using a dental mirror and a periodontal probe. Periodontal examination included plaque scores, calculus scores, probing depth measurements and bleeding on probing. These measurements were recorded for the Ramfjord index teeth.

**Results.** A total of 397 15-year-old participants were examined in this pathfinder study. There were 240 females (60.45%) and 157 males (39.55%). Of the total participants 196 (49.37%) were urban adolescents while 201 (50.63%) were from rural communities. Mean probing depth was 3.34 ± 0.57 mm with a range of 1 to 10 mm; a relatively high proportion (34.26%) of these subjects presented with at least one site with pockets of 5 mm or deeper. Males presented with greater plaque, calculus and probing depths than females. When urban and rural populations were compared, urban participants presented with more plaque, probing depths and bleeding on probing. Greater pocket depths were found to be related to the presence of plaque calculus and bleeding on probing.

**Conclusions.** Overall, rather high incidences of periodontal pockets ≥ 5 mm were detected in this population. This data should serve to prepare further more detailed epidemiological studies that will serve to plan and implement prevent and treat strategies for periodontal diseases in Georgia and also help make manpower decisions.

## Introduction

Epidemiology is the study of the health and disease in populations, as compared to individuals. Periodontal diseases are inflammatory disorders caused by the specific microorganisms in the dental plaque that may lead to loss of periodontal attachment, including destruction of the periodontal ligament and adjacent supporting bone. Periodontal probing depth provides useful information about the present inflammatory status of periodontal tissue, and may also be indicative of the chronicity of the local inflammation (Susin et al., 2005). Significant data on periodontal disease prevalence and distribution among different populations were collected using probing pocket depth (PPD) as the primary variable (Albandar & Rams, 2002).

Population-based studies provide external validity to observations obtained from more discrete subject groups, and enable generalization of the conclusions (Elwood, 1988). Population studies confirmed the close relationship between dental plaque and gingivitis that was initially described by Löe, Theilade & Jensen (1965) in non-population based studies. In the numerous studies worldwide, dental plaque growth and inflammation of gingival tissue are ubiquitous and strongly linked, irrespective of age, gender or racial/ethnic identification (Albandar & Rams, 2002). More than 82% of US adolescents have overt gingivitis and signs of gingival bleeding (Albandar et al., 1998), with similar or higher prevalence of gingivitis being reported for children and adolescents in other parts of the world (Gjermo et al., 2002; Petersen & Kaka, 1999; Levin et al., 2006). Significant disparities appear to exist in the level of periodontitis among young, adult and senior populations in the world. These disparities are also related to different definitions of periodontal diseases, i.e., chronic and aggressive. Subjects of African ethnicity seem to have the highest prevalence of periodontitis, followed by Hispanics and Asians (Albandar & Rams, 2002). A recent survey of Armenian region schoolchildren showed a tendency for an increase in periodontal indices. Periodontal pocket depths of 4–5 mm among these juveniles were reported in 2.04% of the cases (Manrikian, Markarian & Vardanian, 2012).

In a cross-sectional study, the prevalence of aggressive periodontitis among 15 to 18 year-old students in high schools in Tehran, Iran was reported to be only 0.13% (Sadeghi, 2010), though the method used in that study might have caused some underestimation. In another study from Iran, the prevalence of gingivitis was 97% (in 6–7 years), 98.1% (7–8 years), 98.5% (in 8–9 years) and 97.9% in all groups (Jalaleddin & Ramezani, 2009). In a national survey of oral health status of children and adults in Turkey, it was reported that healthy periodontal condition was noted in 56.2% of 15-year-olds. In the 35–44 year-old cohort, calculus prevalence was high (62.6%), and 1.2% had attachment losses of 6 mm or greater which might represent the chronic type of periodontitis (Gökalp et al., 2010).

Epidemiologic data can form the basis for selection and implementation of strategies to prevent and treat periodontal diseases. There are currently no available data regarding the periodontal status of adolescents in Georgia.

Thus the aim of the present pathfinder study was to provide a current periodontal disease mapping of the Georgian population following the guidelines of the World Health Organization for population based surveys.

## Materials and Methods

A pathfinder study was conducted during January to July of 2012, according to the general guidelines of the World Health Organization (1997). IRB Approval for this screening was waived by Tbilisi University. Prior to the study commencement, a training session was performed for the examiners to ensure correct interpretation and mutual agreement on the measurements and indices. Tenth grade school children, 15-year-olds were examined in Tbilisi, as well as in two other large cities (Kutaisi and Batumi) and 4 smaller villages (Akhaltsikhe, Marneuli, Ambrolauri and Tlughi). This study design allowed for the inclusion of the diverse and heterogeneous populations of Georgia. All participants were examined by a trained dental team in a classroom setting using a dental mirror and a University of North Carolina (UNC15) periodontal probe (Hu-Friedy, Chicago, Illinois, USA).

Periodontal examination included (Beltrán-Aguilar et al., 2012; Weinberg & Hassan, 2012):

1. Plaque scores–the presence or absence of visible plaque on any surface of the tooth.
2. Calculus scores–the presence or absence of visible calculus deposits on each tooth.
3. Probing depth–measured from the free gingival margin to the base of the sulcus (in mm).
4. Bleeding on probing–the presence or absence of bleeding following probing around the tooth.

These periodontal measurements were performed for the Ramfjord index teeth (teeth number: 16, 21, 24, 36, 41, 44) which are representative of the various teeth type (Kingman, Susin & Albandar, 2008).

Statistical analysis included descriptive statistics as well as one-way ANOVA with Fisher LSD test and correlation analysis. Statistics were performed using StatPlus 2009 software.

## Results

A total of 397 15-year-old participants were examined periodontally. There were 240 females (60.45%) and 157 males (39.55%). Of these 196 (49.37%) were city dwellers while 201 (50.63%) were from rural areas.

Table 1 depicts the overall periodontal findings in this group. Plaque (on at least one tooth) was detected in most of the participants (73.3%) and calculus in 38.3%. The mean percentile of sites with detectable plaque was 54.81 ± 42.5% (SD) with individual patients mean plaque score ranging from 0 to 100%. The mean percentile of sites with detectable calculus was 12.4 ± 21.5% (SD) with individual patients mean calculus score ranging from 0 to 100%.

**Table 1:** Periodontal findings in the overall group.

Variable	Percentage of participants with positive findings	Mean value (SD)
Plaque	73.3%	54.81% ± 42.5%
Calculus	38.3%	12.38% ± 21.5%
Bleeding on probing	25.94%	5.69% ± 11.6%
Probing depth ( ≥ 5)	34.26%	3.34 ± 0.57

Bleeding on probing was detected in 25.94% of the participants. The mean percentile of sites with bleeding on probing was 5.69 ± 11.6% (SD) with individual patients mean bleeding on probing ranging from 0 to 70%.

**Figure 1:**
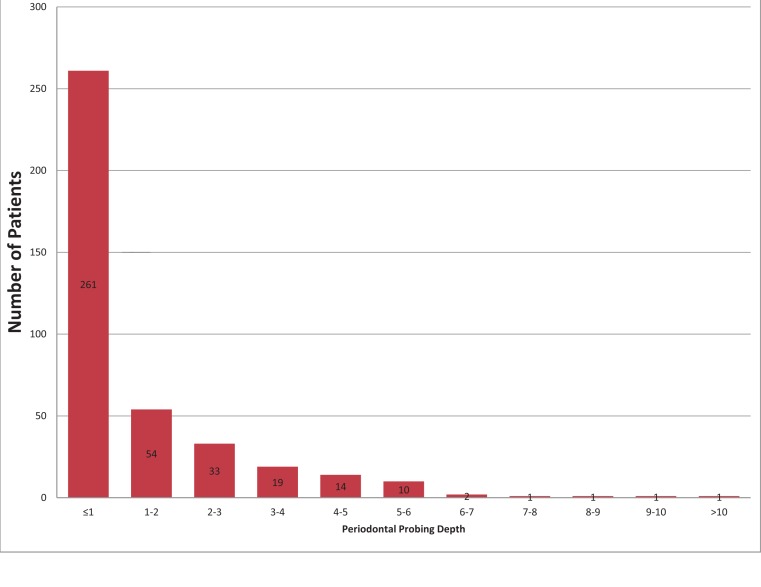
The distribution of the probing depths among study participants.

Mean probing depth was 3.34 ± 0.57 mm with a range of 1 to 10 mm; 34.26% of the participants presented with pockets of 5 mm or deeper. Individual patients mean pocket depth ranged from 1.8 to 5 mm. The distribution of the probing depths is shown on Fig. 1.

Males presented with higher plaque scores (0.67 vs. 0.46 in females; *p* < 0.0001), calculus (0.18 vs. 0.09 in females; *p* = 0.0001) and probing depths (3.42 vs. 3.29 in females; *p* = 0.03) than females (Table 2).

**Table 2:** Comparison between male and female participants.

Variable	Males	Females	*p* value
Plaque	0.67	0.46	<0.0001
Calculus	0.18	0.09	0.0001
Bleeding on probing	0.05	0.06	0.57
Probing depth	3.42	3.29	0.03

When comparing urban and rural population, urban participants presented with more plaque (0.61 vs. 0.49 in rural; *p* = 0.0082), probing depths (3.47 vs. 3.21 in rural; *p* < 0.001) and bleeding on probing (0.072 vs. 0.042 in rural; *p* = 0.0112; Table 3).

**Table 3:** Comparison between urban and rural participants.

Variable	Urban	Rural	*p* value
Plaque	0.61	0.49	0.0082
Calculus	0.14	0.11	0.156
Bleeding on probing	0.072	0.042	0.0112
Probing depth	3.47	3.21	<0.001

Pocket depths were found to be weakly but positively related to the presence of plaque (*r* = 0.3; *p* < 0.001) calculus (*r* = 0.15; *p* = 0.0018) and bleeding on probing (*r* = 0.16; *p* = 0.0021).

## Discussion

This paper presents for the first time updated data for periodontal disease prevalence among adolescents in Georgia. Overall, rather high prevalence of periodontal pockets above 5 mm was detected in this study.

Males in this study had a significantly higher occurrence of high probing depths than females, which is in agreement with findings in other studies (Susin et al., 2005; Morris, Steele & White, 2001). Ericsson et al. (2009) conducted an epidemiological survey aimed at analyzing the periodontal conditions of 19-year-old individuals in two rural counties in Sweden, with special reference to gender and socioeconomic grouping. This study revealed a mean prevalence of sites with probing depth (PPD) of > or = 4 mm to be approximately 8%. Logistic regression analyses revealed that gender (males) and the specific county area were significant factors for a high plaque and gingivitis score.

Urban participants presented with more plaque, probing depths and bleeding on probing. This finding is in contrast to what was reported in the national survey of oral health status of children and adults in Turkey, where rural subjects had more severe periodontal problems than their urban counterparts (Gökalp et al., 2010). Karunachandra et al. found that antenatal women in Sri Lanka have a high burden of dental caries and periodontal disease; prevalence of calculus was 30.3% for rural women and 13.5% for urban women (Karunachandra, Perera & Fernando, 2012). The most significant finding was 3.5% prevalence of shallow periodontal pockets (4–5 mm) for rural women but 73.0% for urban women (*p* = 0.0001). However, in another study (Bonifácio et al., 2011) the prevalence of periodonto-pathogens in a black Brazilian secluded community was found to be similar to black urban population. Those inconsistencies in the findings between rural and urban populations might be attributed to the specific habits and educational levels with regards to oral hygiene as well as to dietary practices.

Population based studies have suggested that good oral hygiene is correlated with a low level of periodontal diseases (Amarasena et al., 2002; Al-Wahadni & Linden, 2003). Likewise, in the present study low levels of plaque and calculus were found to be related to lower probing depths.

Many studies have used the Community Periodontal Index of Treatment Needs (CPITN) or a modification of this index, and most have used convenience study samples. However, the validity of the CPITN as a measure of periodontal status of populations has been questioned (Kingman & Albandar, 2002). Thus, it was suggested to use the prevalence, extent and severity of probing depths as parameters for periodontal health and disease assessment (Susin et al., 2005).

Epidemiologic data, as provided in this report, should serve as a basis for selection and implementation of strategies to prevent and treat periodontal diseases. Improvement of the overall oral hygiene in this population should have a notable impact on the occurrence of periodontal disease. Awareness of the occurrence of disease, the infectious nature of these diseases, and the available means for disease prevention, may be achieved through a better interaction between oral health providers and community decision makers; this should lead to changes in the educational programs to promote healthy attitudes (Albandar & Rams, 2002; Croxson, 1993; Luhanga & Ntabaye, 2001; Levin, 2012).

Destructive forms of periodontal diseases, such as aggressive periodontitis (localized and generalized), usually diagnosed in a young population might not be as rare as they were once believed to be (Levin et al., 2006; Demmer & Papapanou, 2010). The high prevalence of aggressive periodontitis reported among young adolescents as well as our current findings of high prevalence of deep periodontal pockets in 15-year-olds might warrant both pediatric and general dentists to increase their awareness to diagnose the disease as early as possible. Since the disease usually appears at a younger age, the importance of early detection is paramount in order to avoid tooth loss in early childhood or adolescence (Levin et al., 2012; Levin, 2011).

This study has several limitations that should be taken into consideration when evaluating the present results. First, clinical attachment loss (CAL) is considered one of the more common measurements in epidemiological studies to assess the severity of periodontal disease; however, while the use of CAL might have had some advantages, this measure of periodontal disease level is very difficult to perform under these field conditions. Also, these young individuals often present with incomplete passive eruption which might result in misreading of the free gingival margins and consequently CAL. This was the reason to use pocket depth measurement in this study. Second, the utilization of partial examination as in this study is known to cause some underestimation of the overall prevalence of the disease; nevertheless, this is a most commonly used method in epidemiological studies. Furthermore, Kingman et al. have shown a very good correlation between partial and full examinations (Kingman, Susin & Albandar, 2008).

## Conclusion

The prevalence of periodontal diseases in young Georgian population was determined to be relatively high in this pathfinder study. This data, together with more thorough future epidemiological studies, should serve to plan treatment needs and manpower allocation in Georgia.
